# Association of Cardiovascular Biomarkers With Cardiac Allograft Vasculopathy and Atherosclerotic Coronary Artery Disease

**DOI:** 10.1111/ctr.70195

**Published:** 2025-06-09

**Authors:** Lukas Köster, Jessica Weimann, Alexander Bernhard, Benjamin Bay, Christopher M. Blaum, Thiess Lorenz, Tanja Zeller, Christoph Waldeyer, Hermann Reichenspurner, Paulus Kirchhof, Stefan Blankenberg, Christina Magnussen, Fabian J. Brunner

**Affiliations:** ^1^ Department of Cardiology University Heart & Vascular Center Hamburg University Medical Center Hamburg‐Eppendorf Hamburg Germany; ^2^ German Center for Cardiovascular Research (DZHK) Partner Site Hamburg/Kiel/Lübeck Hamburg Germany; ^3^ Department of Cardiovascular Surgery University Heart and Vascular Center Hamburg University Medical Center Hamburg Germany; ^4^ Center for Population Health Innovation (POINT Institute) University Heart and Vascular Center Hamburg University Medical Center Hamburg‐Eppendorf Hamburg Germany; ^5^ Institute of Cardiovascular Sciences University of Birmingham Birmingham UK

**Keywords:** biomarkers, brain, cardiac allograft vasculopathy, coronary artery disease, C‐reactive protein, heart transplantation, natriuretic peptide, troponin I, troponin T

## Abstract

Background: Cardiac allograft vasculopathy (CAV) remains a barrier to long‐term survival after heart transplantation. Little is known about cardiovascular biomarkers in CAV and how they compare to biomarkers in atherosclerotic coronary artery disease (CAD).

Purpose: This study addresses these gaps by investigating the associations of high‐sensitivity troponin I and T (hsTnI/T), N‐terminal pro‐B‐type natriuretic peptide (NT‐proBNP) and high‐sensitivity C‐reactive protein (hsCRP) with CAV and CAD.

Methods: Posttransplant patients undergoing angiography were matched 1:2 with nontransplant patients by age, sex, hypertension, BMI and angiographic severity of CAV and CAD. Disease severity was classified using the International Society for Heart and Lung Transplantation.

Results: Sixty‐three transplant and 126 matched nontransplant patients (median age 55.9 years, 5.8 years posttransplant, 8.5% female) were analyzed. Among transplant patients, 17.5% had mild, 7.9% moderate, 7.9% severe, and 66.7% no CAV. HsTnI/T (OR per SD = 2.21/2.38, CI = 1.17–4.66/1.21–5.69, *p* = 0.022/0.026) and NT‐proBNP (OR per SD = 2.86, 95% CI 1.50–6.39, *p* = 0.004) were significantly associated with CAV. While hsTnT (OR per SD = 1.60, 95% CI 1.06–2.52, *p* = 0.030) and hsCRP (OR per SD = 1.61, 95% CI 1.08–2.47, *p* = 0.023) were significantly associated with CAD.

Conclusion: Distinct biomarker profiles were observed: HsTnI, hsTnT, and NT‐proBNP showed associations with CAV, while hsTnT and hsCRP were associated with CAD.

**Abbreviations**
CAD: coronary artery disease
CAV: cardiac allograft vasculopathy
CI: confidence interval
CMV: cytomegalovirus
eGFR: estimated glomerular filtration rate
HbA1c: hemoglobin A1c
hsCRP: high‐sensitivity C‐reactive protein
hsTn: high‐sensitivity troponin
hsTnI: high‐sensitivity troponin I
hsTnT: high‐sensitivity troponin T
HTX: heart transplantation
IQR: interquartile range
ISHLT: International Society for Heart and Lung Transplantation
LDL‐C: low‐density lipoprotein cholesterol
LM: left main
NT‐proBNP: N‐terminal pro‐B‐type natriuretic peptide
OR: odds ratio
SD: standard deviation
TG: triglycerides
VD: vessel disease

## 1 Introduction

Cardiac allograft vasculopathy (CAV) is one of the leading causes of graft failure after heart transplantation (HTX) [1]. The development of CAV is influenced by immunological and non‐immunological factors and shares several parallels with atherosclerotic coronary artery disease (CAD) [2, 3, 4, 5]. Both conditions are characterized by the progressive narrowing, rarefaction, and stenosis of the coronary arteries based on inflammation, smooth muscle cell proliferation, and atherogenesis within the coronary wall [2, 3, 4, 5, 6, 7, 8]. In particular, CAV features concentric intimal thickening of all epicardial vessels, including the intramuscular arteries and the microvascular bed [2, 4, 5]. This results in a diffuse disease contrasting the typically focal phenotype in CAD [2, 3, 4, 5, 6, 8]. Specific therapeutic options for CAV are limited compared to CAD [2, 4, 5, 6]. As such, interventional and surgical revascularization procedures have not proven to enhance overall survival and retransplantation is rarely feasible [2, 4, 5, 6, 9, 10, 11]. Primary and secondary prevention strategies, including intensified immunosuppression regimens, are the main methods to reduce disease incidence and slow progression. These strategies often lead to severe adverse effects [2, 4]. Consequently, early risk stratification is essential. While previous studies have partly explored high‐sensitivity troponin I and T (hsTnI/T), N‐terminal pro‐B‐type natriuretic peptide (NT‐proBNP) and high‐sensitivity C‐reactive protein (hsCRP) individually in this context, our study provides a direct comparative analysis of their associations with CAV and CAD [12, 13, 14, 15, 16, 17, 18, 19]. Despite growing interest in novel biomarkers, myocardial imaging, and coronary physiology measurements, we hypothesize that these established cardiovascular biomarkers remain clinically relevant, as the different pathophysiological mechanisms of CAV and CAD likely produce distinct profiles. Therefore, this study aims to directly compare the associations of hsTnI/T, NT‐proBNP, and hsCRP with CAV and CAD severity in a matched cohort.

## 2 Methods

### 2.1 Study Cohort

**FIGURE 1:**
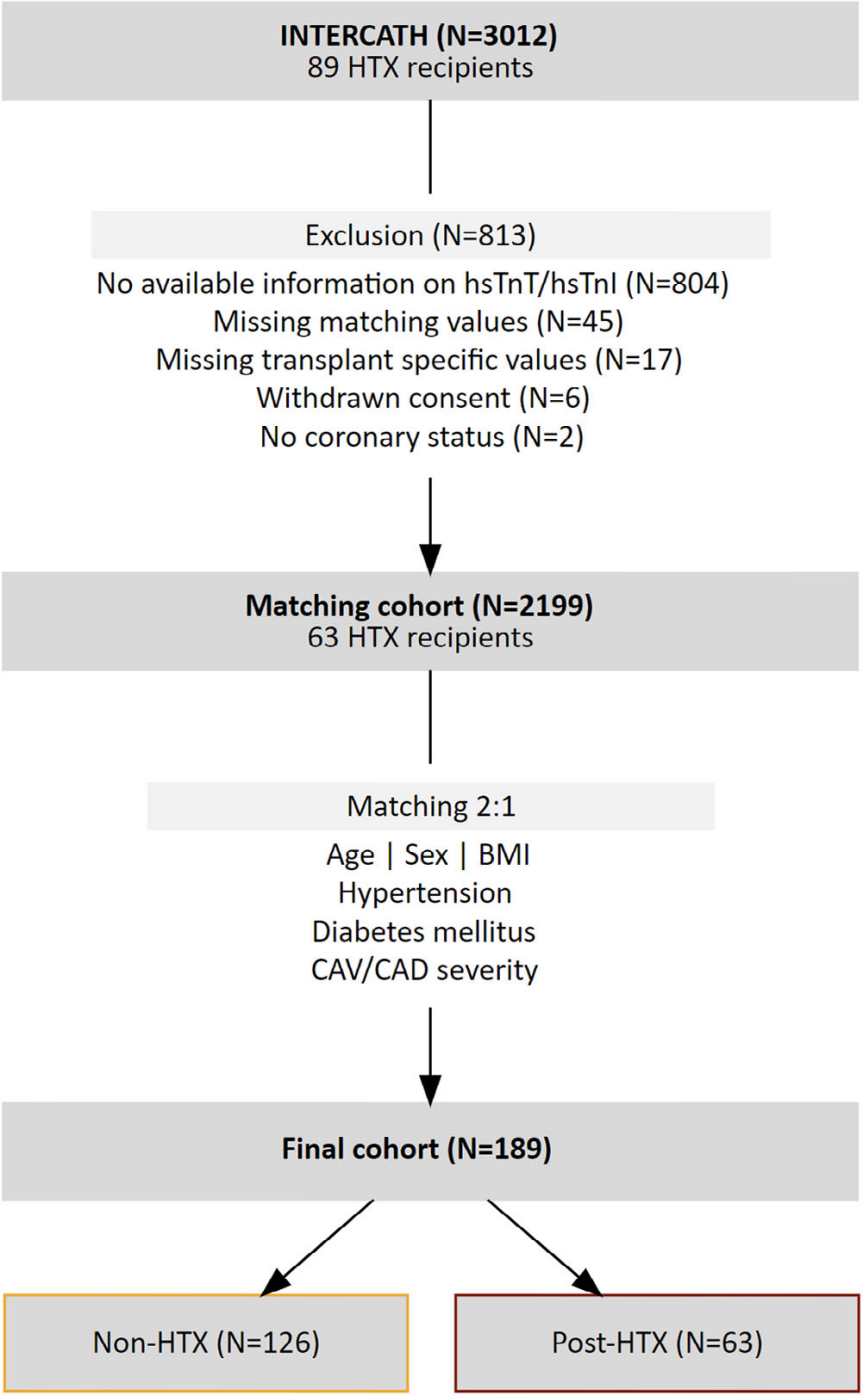
Selection, exclusion, and matching of participants. The initial cohort underwent an exclusion process, followed by the matching of HTX recipients with non‐NTX patients, culminating in the creation of the final cohort. Some patients met more than one reason for exclusion. BMI = body mass index, CAD = coronary artery disease, CAV = cardiac allograft vasculopathy, hsTnI = high‐sensitivity troponin I, hsTnT = high‐sensitivity troponin T, HTX = heart transplantation.

The INTERCATH study is a German single‐center prospective cohort study initiated in 2015. Rationale and design are accessible at clinicaltrials.gov (NCT04936438) and have been described in detail before [20, 21]. In brief, patients undergoing coronary angiography at the University Heart and Vascular Centre of the University Medical Centre Hamburg‐Eppendorf, Germany, were included. The study is approved by the Ethics Committee of Hamburg, Germany (PV4303), and written informed consent of each participant was given. A total of 3012 patients were recruited from 2015 to 2021. Only clinically stable patients, as assessed by the treating physician, were included at the time of study participation. No explicit exclusion criteria were applied regarding recent rejection or prior graft dysfunction. Both hsTnI and hsTnT were measured in 2244 patients. Patients without those measurements were excluded. Further exclusions, as detailed in Figure 1, resulted in a cohort of 2199 patients including 63 HTX recipients. The 63 HTX recipients were matched in a 1:2 ratio with 126 non‐HTX patients, forming the final cohort of 189 individuals for analysis. Details and matching criteria are provided in the statistics section below.

### 2.2 CAV and CAD Grading

For CAV, a team of two experienced interventional cardiologists and the first author of the present study assessed digitally recorded angiograms. In accordance with the consensus nomenclature for CAV by the International Society for Heart and Lung Transplantation (ISHLT) the extend of CAV was angiographically graded as CAV 0 (Not significant): No detectable angiographic lesion; CAV 1 (Mild): Angiographic left main (LM) < 50%, or primary vessel with maximum lesion of < 70%, or any branch stenosis < 70%; CAV 2 (Moderate): Angiographic LM < 50%; a single primary vessel ≥ 70%, or isolated branch stenosis ≥ 70% in branches of 2 systems; CAV 3 (Severe): Angiographic LM ≥ 50%, or two or more primary vessels ≥ 70% stenosis, or isolated branch stenosis ≥ 70% in all 3 systems [22]. For CAD the grading was performed by experienced interventional cardiologists and categorized in CAD 0 (Not significant), 1‐VD (One‐vessel disease), 2‐VD (Two‐vessel disease), and 3‐VD (Three‐vessel disease) according to the number of affected main coronary vessels with ≥50% stenosis. At our center, routine coronary angiography is performed one year post‐transplant and every two years thereafter or earlier if clinically indicated.

### 2.3 Medication and Medical History

Standardized questionaries and medical records were used to obtain current medication and medical history at baseline. Hypertension was defined as a documented diagnosis of hypertension, self‐reported diagnosis of hypertension or the use of antihypertensive medication. Diabetes mellitus was identified through self‐reported diagnosis of diabetes mellitus, documented diagnosis of diabetes mellitus, intake of antidiabetic medication or a hemoglobin A1c (HbA1c) > 6.5%. Hyperlipoproteinemia was determined by either self‐reported or documented diagnosis of dyslipidemia or use of lipid‐lowering medications.

### 2.4 Laboratory Methods

Routine laboratory methods were applied to determine low‐density lipoprotein cholesterol (LDL‐C), triglycerides (TG), estimated glomerular filtration rate (eGFR), HbA1c, and NT‐proBNP. Troponin concentrations were determined via commercially available immunoassays for hsTnI (Abbott Diagnostics, ARCHITECT STAT) and hsTnT (Roche Diagnostics Elecsys). The lower limits of detection were reported as 1.9 ng/L for hsTnI and 5 ng/L for hsTnT. HsCRP was measured with the CardioPhase hsCRP assay (Siemens Healthcare Diagnostics Products) and the lower limit of detection was reported as 0.01 mg/L.

### 2.5 Statistical Analysis

Categorical variables were reported as absolute numbers and percentages and compared using the chi‐square test. Continuous variables were described as median with the 25th and 75th percentile and compared using the Mann–Whitney test. Nearest neighbor matching was conducted in a 1:2 fashion using age, sex, hypertension, body mass index (BMI), diabetes mellitus. The severity of CAV or CAD was matched in an exact technique. Propensity score matching was used to compare post‐HTX and non‐HTX patients to account for differences between groups. The average absolute standardized difference was 0.52 before matching and 0.04 after matching. The median time since transplantation was calculated by reverse Kaplan–Meier estimator. To evaluate associations between hsTnI/T, NT‐proBNP, and hsCRP with CAV and CAD, univariable logistic regression analyses were conducted. Due to their non‐normal distribution, the dependent variables, hsTnI/T, NT‐proBNP, and hsCRP, were log‐transformed (log) and reported accordingly. For categorical variables, Odds Ratio (OR) and for the continuous variables OR per standard deviation (SD) were calculated and reported. Adjusted regression analysis for sex and age were conducted to account for confounders. Statistical significance was determined at a two‐sided alpha level of <0.05. All statistical analyses were carried out using the R statistical software version 4.0.3 and “MatchIt”‐package (2010‐10‐10, R Foundation for Statistical Computing).

## 3 Results

### 3.1 Baseline Characteristics

A total of 189 patients, with 63 HTX recipients and 126 patients in the matched non‐HTX cohort, were analyzed. Detailed patient characteristics are displayed in Table 1. Median age was 55.9 years (Interquartile range (IQR) 48.1–65.7) and 8.5% of the participants were women. A higher prevalence of dyslipidemia was observed in the HTX group (73.0% vs. 34.7%, *p* < 0.001), along with elevated triglyceride blood levels (215 mg/dL vs. 121 mg/dL, *p* < 0.001). Median baseline LDL‐C was 91 mg/dL, with no significant difference between both groups. Renal function was impaired in patients after HTX, as reflected by reduced eGFR (47.3 mL/min vs 81.9 mL/min, *p* < 0.001). Other cardiovascular risk factors and laboratory parameters did not differ significantly between groups. HTX recipients specific, the median time since transplantation was 7.0 years (95% Confidence Interval (CI) 4.7–9.3) and immunosuppressive therapy consisted of mammalian target of rapamycin inhibitors in 76.3%, calcineurin inhibitors in 72.4% and steroids in 71.2% of recipients.

**TABLE 1:** Baseline patient characteristics.

Patient characteristics	All (*N* = 189)	Non‐HTX (*N* = 126)	Post‐HTX (*N* = 63)	*p*
Age, years	55.9 (48.1, 65.7)	55.8 (48.1, 65.7)	56.1 (48.1, 65)	0.84
Female (%)	16 (8.5)	11 (8.7)	5 (7.9)	1.00
**Cardiovascular RF**				
BMI	26.9 (24, 30.1)	26.9 (24.3, 30)	26.8 (23.7, 30.6)	0.89
Hypertension (%)	138 (73.0)	91 (72.2)	47 (74.6)	0.86
Dyslipidemia (%)	89 (47.6)	43 (34.7)	46 (73)	<0.001
Diabetes mellitus (%)	46 (24.3)	31 (24.6)	15 (23.8)	1.00
Current smoking (%)	33 (18.2)	27 (22.5)	6 (9.8)	0.06
**Laboratory parameters**				
LDL‐C (mg/dL)	91 (73.2, 118.8)	93 (73.7, 116.3)	87.5 (73, 131.3)	0.80
Triglycerides (mg/dL)	132.5 (94.4, 219.1)	121 (86, 163)	215 (140.3, 305.3)	<0.001
Creatinine (mg/dL)	1.1 (0.9, 1.4)	1.0 (0.8, 1.2)	1.5 (1.2, 2)	<0.001
eGFR (mL/min)	74.4 (52.8, 91.5)	81.9 (65.4, 96.7)	47.3 (36, 72.1)	<0.001
HbA1c (%)	5.6 (5.2, 6.3)	5.5 (5.2, 6.3)	5.7 (5.3, 6.4)	0.41
hsTnI (ng/L)	5.6 (2.5, 14)	5.8 (2.4, 14.3)	5.1 (2.5, 11.3)	0.74
hsTnT (ng/L)	14.0 (7, 24.3)	12.5 (7, 24.1)	17.0 (9.2, 26.5)	0.07
NTproBNP (pg/mL)	480 (127.2, 1745.8)	379 (93.1, 1679.6)	627 (244, 1912.7)	0.06
hsCRP (mg/L)	0.4 (0.1, 0.9)	0.4 (0.1, 1)	0.3 (0.2, 0.8)	0.67
**HTX‐specific**				
Age at HTX (years)	—	—	50.8 (43.8, 58.1)	—
Pre‐HTX heart surgery (%)	—	—	8 (26.7)	—
CMV mismatch (D+/R−, %)	—	—	10 (23.3)	—
History of rejection	—	—	3 (4.8)	—
History of PGD	—	—	4 (6.3)	—
**HTX indication**				
DCM (%)	—	—	28 (47.5)	—
H(O)CM (%)	—	—	2 (3.4)	—
ICM (%)	—	—	20 (33.9)	—
Other (%)	—	—	9 (15.3)	—
**Immunosuppressants**				
Steroids (%)	—	—	42 (71.2)	—
Calcineurin inhibitors (%)	—	—	42 (72.4)	—
Cyclosporine (%)	—	—	10 (16.9)	—
Tacrolimus (%)	—	—	32 (55.2)	—
mTOR‐inhibitors (%)	—	—	45 (76.3)	—
Everolimus (%)	—	—	45 (76.3)	—
Sirolimus (%)	—	—	0 (0)	—
MMF (%)	—	—	28 (47.5)	—

*Note:* Displayed are baseline characteristics of the study participants, divided into three groups: All participants, non‐heart transplant patients and post‐heart transplant patients. *p* values are shown on the right for comparison between groups. Age and Immunosuppressant use were measured at the time of baseline. History of rejection is defined as any biopsy‐proven rejection ≥ ISHLT grade 2R. Data presented as median (interquartile range) or number (%).

Abbreviations: BMI = body mass index, CMV = cytomegalovirus, DCM = dilated cardiomyopathy, eGFR = estimated glomerular filtration rate, H(O)CM = hypertrophic (obstructive) cardiomyopathy, HbA1c = hemoglobinA1c, hsCRP = high‐sensitivity C‐reactive protein, hsTnI = high‐sensitivity troponin I, hsTnT = high‐sensitivity troponin T, HTX = heart transplantation, ICM = ischemic cardiomyopathy, IQR = interquartile range, LDL‐C = low‐density lipoprotein cholesterol, MMF = mycophenolate mofetil, mTOR = mammalian target of rapamycin, NTproBNP = N‐terminal pro‐brain natriuretic peptide, PGD = primary graft dysfunction, RF = risk factors.

### 3.2 Pretransplantation Characteristics

The median age at transplantation was 50.8 years (IQR 43.8–58.1). Pretransplant major heart surgeries were conducted in 26.7% of the patients. Cytomegalovirus (CMV) mismatch (D+/R−) was evident in 23.3% of the recipients. The leading reason for transplantation was dilated cardiomyopathy (47.5%), followed by ischemic cardiomyopathy (33.9%), hypertrophic cardiomyopathy (3.4%), and other causes (15.3%).

### 3.3 CAV and CAD Prevalence

**FIGURE 2:**
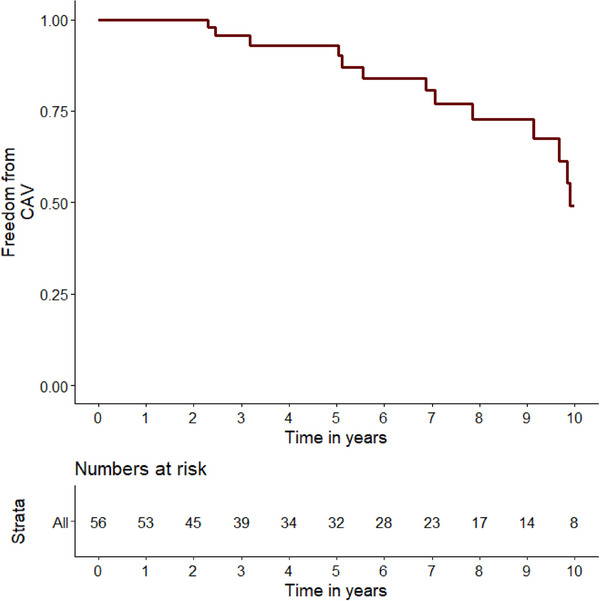
Freedom from cardiac allograft vasculopathy after heart transplantation. Cumulative freedom from CAV in survivors 1–10 years after HTX. CAV = cardiac allograft vasculopathy, HTX = heart transplantation.

Freedom from CAV was 93.0% at 5 years and 49.1% at 10 years after transplantation estimated by Kaplan–Meier method (Figure 2). At a median of 7.2 years post‐transplantation, 66.7% of HTX recipients were categorized as CAV0. Eleven (17.5%), 5 (7.9%), and 5 (7.9%) were diagnosed with CAV grades 1, 2, and 3. Since both groups were matched for disease severity, identical percentages were observed for CAD0 (66.7%), 1‐, 2‐, and 3‐VD with 22 (17.5%), 10 (7.9%) and 10 (7.9%) (Table S1 and Figure S3).

### 3.4 Association of hsTnI/T, NT‐proBNP, and hsCRP With CAV and CAD

**FIGURE 3:**
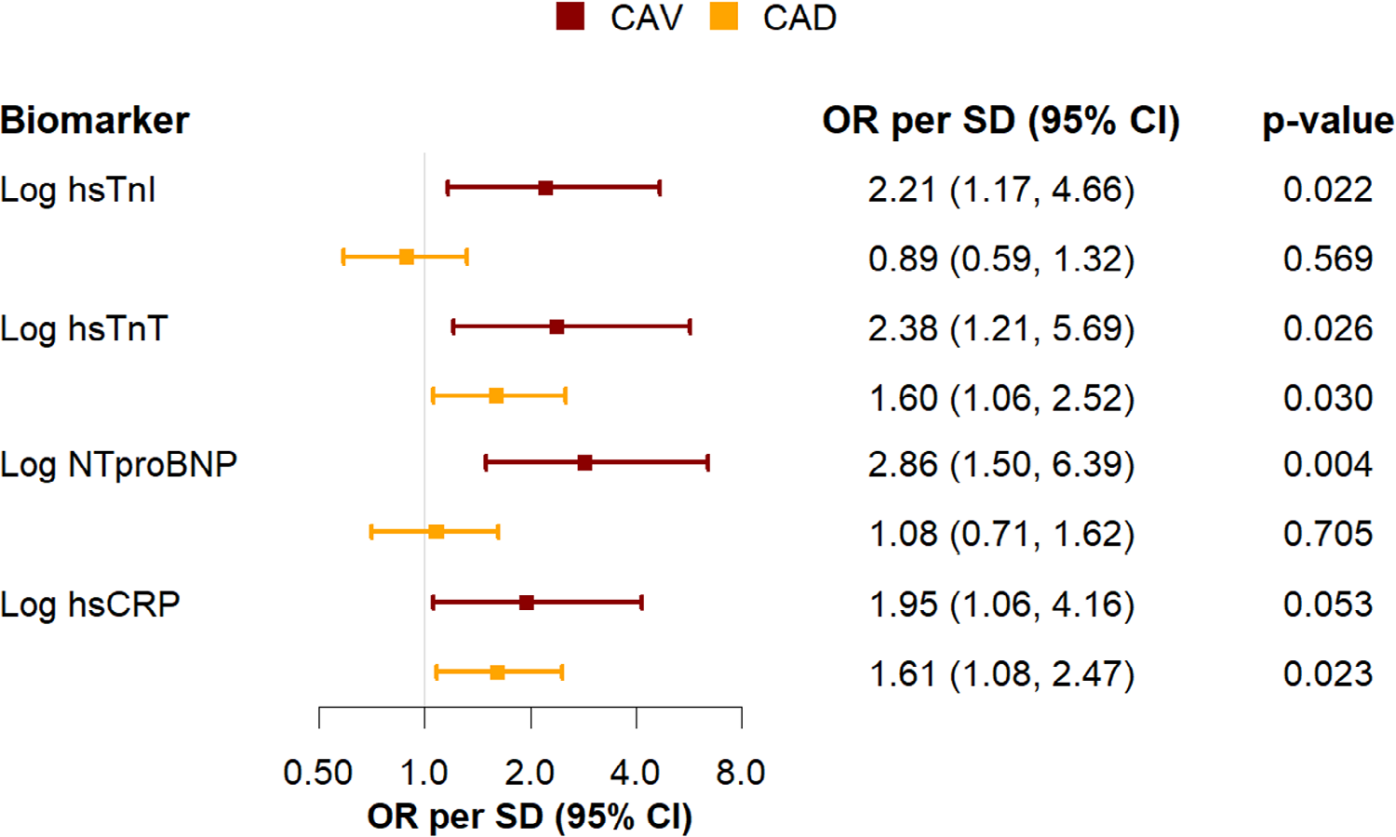
Logistic regression investigating the association of biomarkers with cardiac allograft vasculopathy and coronary artery disease adjusted by sex and age. Odds ratio per standard deviation and their 95‐confidence interval are provided. Biomarkers are presented log‐transformed. CAD = coronary artery disease, CAV = cardiac allograft vasculopathy, hsTnT = high‐sensitivity troponin T, hsTnI = high‐sensitivity troponin I, hsCRP = high‐sensitivity C‐reactive PROTEIN, NT‐proBNP = N‐terminal pro‐B‐type natriuretic peptide, OR = odds ratio, SD = standard deviation.

Adjusted for age and sex, significant associations for _log_hsTnI (Odds Ratio per standard deviation (OR) = 2.21, 95% CI: 1.17–4.66, *p* = 0.022), _log_hsTnT (OR = 2.38, 95% CI: 1.21–5.69, *p* = 0.026), and _log_NT‐proBNP (OR = 2.86, 95% CI: 1.50–6.39, *p* = 0.004) with CAV were documented. The association of _log_hsCRP (OR = 1.95, 95% CI: 1.06–4.16, *p* = 0.053) with CAV did not reach the predefined significance level (Figure 3). For CAD, significant associations were found for _log_hsTnT (OR 1.60, 95% CI: 1.06–2.52, *p* = 0.030) and _log_hsCRP (OR = 1.61, 95% CI: 1.08–2.47, *p* = 0.023), but not for _log_hsTnI (OR 0.89, 95% CI: 0.59–1.32, *p* = 0.569) and _log_NT‐proBNP (OR 1.08, 95% CI: 0.71–1.62, *p* = 0.705). Details concerning the associations of cardiovascular risk factors, transplantation‐specific characteristics and laboratory measurements with CAV and CAD are provided in Table S2.

### 3.5 Discussion

This study aimed to examine and compare the associations of hsTnI/T, NT‐proBNP, and hsCRP with the severity of CAV and CAD. The main findings are:

1. HsTnI, hsTnT, and NT‐proBNP showed significant associations with CAV.
2. HsTnT and hsCRP were significantly associated with CAD.
3. The biomarker profile differed distinctly between the two conditions.

### 3.6 Study Cohort

The prevalence of cardiovascular risk factors in our cohort aligns with previous reports on patients who have undergone heart transplantation [23]. Likewise, we observed a high incidence of obesity, hypertension, dyslipidemia, diabetes mellitus, and impaired renal function, including confirmations of significant associations between elevated LDL‐C and reduced eGFR with CAV in the supplemental data. This supports that these conditions are not only common after HTX but are also worsened by the intrinsic stressors of the transplantation process, including surgical trauma, perioperative complications, physical inactivity, and long‐term immunosuppressive therapy. This exacerbation seems to play a critical role in the disease progression, as cardiovascular risk factors and the discussed comorbidities are associated with vascular damage, inflammation, atherosclerotic changes and ultimately with CAV [4, 23, 24].

The proportion of female HTX recipients was lower than expected [1]. This underrepresentation could reflect inclusion bias, as participants were only included when coronary angiography was done. Outside of HTX, women generally undergo coronary angiography less frequently than men [25]. The lower rates of early rejection and CAV in women might nave further reduced angiography frequency and therefore inclusion. This sex imbalance may limit the generalizability of our findings, particularly in understanding potential sex‐specific differences in CAV progression and biomarker associations.

Otherwise, key metrics of our study population—age at transplantation, prevalence of CMV mismatch, time since HTX and reasons for HTX—are consistent with other HTX cohorts, enhancing the representativeness and generalizability of our findings [23, 26].

### 3.7 Prevalence of CAV

Our study found that ten years post‐heart transplantation, 50.9% either developed CAV or died, consistent with international registry data. This occurred despite 70% of patients being treated with mTOR‐Inhibitors, which are typically linked to lower CAV rates. The lack of reduced CAV prevalence in our cohort may be attributed to factors such as mTOR‐Initiation timing, clinical indications for switching or underlying patient risk profiles. However, we observed a lower‐than‐expected prevalence of CAV two and five years after transplantation. This is likely due to temporal selection bias, as INTERCATH began including patients in 2015, which for some patients was years after transplantation, thereby shifting the observations of CAV to later time points. Local practices for prevention and screening, which might differ from international approaches, could partially account for the observed differences. Generally, the prevalence of CAV may be underestimated since the ISHLT classification does not account for microvascular dysfunction, a component of CAV.

### 3.8 Biomarkers

We observed distinct associations of biomarkers with CAV and CAD, emphasizing the underlying pathophysiological differences between these conditions. The differing association of hsTnI/T, NTproBNP, and hsCRP in the two disease entities emphasize the different pathophysiological pathways which are implicated in the progression of CAV and CAD.

High‐sensitivity troponins (hsTns) are well known markers for myocardial injury [27]. For hsTnI, we found significant associations with CAV but not with CAD. This contrasts with previous studies reporting an association between hsTnI and CAD but agrees with emerging evidence that CAV, characterized by diffuse vasculopathy, causes more widespread myocardial injury, potentially causing a pronounced troponin release compared to the typical focal ischemia in CAD [2, 3, 4, 6, 8, 28, 29, 30, 31]. The lack of association of hsTnI with CAD in our study may reflect the small sample size and the absence of more granular CAD scoring systems, such as SYNTAX or Gensini scores, which might have captured subtle associations observed in previous research. In contrast, hsTnT was significantly associated with both CAV and CAD, extending the limited knowledge of its relevance to CAV. These divergent associations between the different troponin subtypes may reflect subtle pathophysiological and biochemical properties of these markers. HsTnI, with faster release kinetics, may be particularly sensitive to acute myocardial injury from diffuse processes like CAV. Both markers perform similarly in diagnosing myocardial infarction, though they exhibit variability in chronic or milder conditions. In contrast, hsTnT, with its longer half‐life and biphasic release, remains detectable during prolonged or recurrent stress, which can occur in both CAV and CAD [32]. These differences highlight the complexity of interpreting troponin elevations in HTX recipients, where factors such as allograft rejection, myocardial hypertrophy and renal dysfunction modulate troponin release. But, troponin elevations persist in CAD despite impaired renal function, emphasizing their utility for CAV risk assessment [28, 29, 30, 33]. Given the observed differences, hsTnI and hsTnT provide complementary insights into myocardial injury, reflecting their distinct preanalytical and physiological properties. Their clinical availability and routine use underscore their diagnostic relevance. A targeted approach that accounts for these differences may optimize their application in clinical practice.

Focusing on NT‐proBNP, we observed a significant association with CAV, which is in concordance with previous research which documented the association of NT‐proBNP with adverse outcomes in HTX patients, including graft dysfunction, rejection, major adverse cardiovascular events and, in two studies from the early 2000s conducted prior to the establishment of the ISHLT consensus nomenclature, with CAV [12, 13, 18]. Cytokine‐mediated upregulation of natriuretic peptides and myocardial strain from microvascular dysfunction, a known CAV pathology, in addition to the well‐established role of NT‐proBNP in the assessment of heart failure, and its release in response to myocardial stretch myocardial stretch, might contribute to the pronounced association of this natriuretic peptide with CAV [34]. Comparable to hsTns, NT‐proBNP lacks specificity as it may also reflect other post‐transplant complications such as heart failure and renal dysfunction. But the strong association supports its relevance as a biomarker for CAV.

Lastly, hsCRP showed a more robust association with CAD in contrast to the association observed with CAV, which for CAD is consistent with its demonstrated importance in the pathophysiology of atherosclerosis and systemic inflammation [35, 36]. For CAV, the observed association approached the predefined statistical threshold for significance (*p* = 0.053) and limited prior research suggests its potential as a marker for allograft vasculopathy that warrants further investigation [37, 38]. The persistent low‐grade inflammation typical of CAV could drive hsCRP elevation, yet the mechanisms and extent seem to differ, with hsCRP lacking the strength of association observed with the traditional atherosclerotic processes seen in CAD. If hsCRP proves to be causative in disease progression, interventions such as adherence to a Mediterranean diet would be recommended and medications aimed at lowering hsCRP should be considered [21, 39].

Building on the strengths, limitations and distinctions observed, our findings highlight the complexity of CAV diagnosis. Each biomarker offers valuable insights but is limited by confounding factors, especially in HTX recipients with their multifactorial disease burden. This challenge has driven research toward novel biomarkers for CAV, though many remain unavailable for routine clinical use. A multi‐marker approach, integrating established biomarkers, could hold the potential to improve diagnostic and prognostic value and capture subtle disease patterns more effectively than individual markers. Future research should focus on refining a composite biomarker profile by integrating additional markers, determining optimal thresholds and validating their utility in larger cohorts. Such efforts could enable earlier detection, guide targeted therapies, and improve long‐term outcomes for HTX recipients [40].

### 3.9 Limitations

Whilst we report from a well characterized contemporary cohort of HTX and matched non‐HTX patients, some limitations must be considered. First, the sample size of the study limits statistical power and generalizability. However, these limitations are mitigated by the thorough characterization of patients through coronary angiography by a defined and trained assessment team and adherence to ISHLT classification, ensuring a precise and standardized approach despite its limitations in capturing microvascular disease, an established component of CAV pathophysiology. The exclusion of HTX patients due to missing data points might introduce bias. But those exclusions are essential for the meticulous matching of patients, thereby minimizing confounding variables. The intentional non‐use of specific CAD classification scores like SYNTAX or Gensini, stems from our study's rigorous adherence to the ISHLT nomenclature that does align better with the number of affected coronary vessels approach we took. This decision ensures interstudy comparability. While being gold standard, intimal hyperplasia, vascular remodeling and microvascular involvement can be underestimated with angiographic grading [22]. Not using intravascular ultrasound, myocardial PET imaging or coronary physiology measurements like index of microcirculatory resistance enhanced practicality and relevance for a broader clinical context but reduced diagnostic accuracy. Biomarker levels may have been influenced by prior rejection or graft dysfunction, which were not exclusion criteria. While this may impact interpretability, it reflects routine clinical practice and enabled inclusion of the full spectrum of disease phenotypes. The small sample size, especially in high‐grade CAV groups, limited reliable subgroup analyses. This reflects both the low prevalence of advanced CAV and the constrained pool of transplant recipients in our single‐center cohort, making targeted enrichment unfeasible. Larger, multicenter studies will be essential to validate these findings and investigate biomarker patterns in advanced CAV. Lastly, due to the observational, cross‐sectional design, longitudinal associations between biomarker levels and CAV or CAD progression could not be evaluated and no cause‐effect relations can be deduced. Despite that, the study design ensures a focused and comparable approach to an important clinical problem focusing on early detection of CAV with widely available diagnostic methods [23].

## 4 Conclusion

This study evaluates the associations between hsTnI, hsTnT, NT‐proBNP, and hsCRP with CAV, compared to CAD, in a well‐matched heart transplant cohort. It identifies distinct biomarker profiles, with hsTnI, hsTnT, and NT‐proBNP being significantly associated with CAV and hsTnT as well as hsCRP with CAD, reflecting the differing pathophysiological mechanisms of the two conditions. Our findings add to the growing body of evidence which investigates the association of biomarkers with CAV, highlighting the potential of a multi‐marker approach to assess patients with and without CAV after HTX. Larger studies are needed to validate these results and refine biomarker‐based strategies for early detection to improve long‐term survival after heart transplantation.

## Conflicts of Interest

The authors declare no conflicts of interest.

## Supporting information




**Table S1**: Grading of Cardiac Allograft Vasculopathy and Coronary Artery Disease Severity. **Table S2**: Associations of Cardiovascular Risk Factors, Transplantation‐Specific Characteristics and Laboratory Measurements with CAV and CAD. **Figure S3**: Prevalence of Coronary Artery Disease and Cardiac Allograft Vasculopathy Severity.

## Data Availability

The data that support the findings of this study are available on request from the corresponding author. The data are not publicly available due to privacy or ethical restrictions.
