# Supplementary material for: Association of Cardiovascular Biomarkers With Cardiac Allograft Vasculopathy and Atherosclerotic Coronary Artery Disease
Source: Clin Transplant. 2025 Jun 9;39(6):e70195. doi: 10.1111/ctr.70195 (PMC12146798; doi:10.1111/ctr.70195)
Supplement: Supplementary file 1 — Table S1: Grading of Cardiac Allograft Vasculopathy and Coronary Artery Disease Severity. Table S2: Associations of Cardiovascular Risk Factors, Transplantation‐Specific Characteristics and Laboratory Measurements with CAV and CAD. Figure S3: Prevalence of Coronary Artery Disease and Cardiac Allograft Vasculopathy Severity. [file CTR-39-e70195-s001.docx]

**Table S1: Grading of Cardiac Allograft Vasculopathy and Coronary Artery Disease Severity.**

| **Grade** | **N = 189** |
| --- | --- |
| CAV | N = 63 |
| No CAV (%) | 42 (66.67) |
| ISHLT Grade 1 (%) | 11 (17.46) |
| ISHLT Grade 2 (%) | 5 (7.94) |
| ISHLT Grade 3 (%) | 5 (7.94) |
| CAD | N = 126 |
| CAD 0 (%) | 84 (66.67) |
| 1-VD (%) | 22 (17.46) |
| 2-VD (%) | 10 (7.94) |
| 3-VD (%) | 10 (7.94) |

The consensus nomenclature for cardiac allograft vasculopathy (CAV) established by the international society for heart and lung transplantation (ISHLT) is used to categorize the severity for CAV^21^. Coronary artery disease (CAD) is categorized in no significant coronary artery disease, 1-, 2-, or 3-vessel disease according to the number of effected major coronary vessels with ≥50% stenosis. Abbreviations: 1-VD = One vessel disease, 2-VD = Two vessel disease, 3-VD = Three vessel disease, CAD = coronary artery disease, CAV = cardiac allograft vasculopathy, CAV 1 = Mild, CAV 2 = Moderate, CAV 3 = Severe, CAD 0 = No Significant Coronary Artery Disease.

**Table S2: Associations of Cardiovascular Risk Factors, Transplantation-Specific Characteristics and Laboratory Measurements with CAV and CAD.**

| **Patient characteristics** | **OR (95% CI)** | **p** |
| --- | --- | --- |
| CAV |  |  |
| Age | 1.01 (0.59, 1.74) | 0.98 |
| BMI | 0.97 (0.56, 1.64) | 0.90 |
| Hypertension | 1.26 (0.74, 2.32) | 0.42 |
| Dyslipidemia | 0.95 (0.57, 1.63) | 0.84 |
| Diabetes mellitus | 1.62 (0.97, 2.75) | 0.07 |
| Current Smoking | 0.73 (0.30, 1.30) | 0.35 |
| LDL-C | 2.19 (1.14, 5.02) | 0.038 |
| eGFR | 0.27 (0.10, 0.57) | 0.002 |
| HbA1c | 1.42 (0.83, 2.55) | 0.20 |
| Age at HTX | 0.81 (0.45, 1.44) | 0.47 |
| Pre-HTX major heart surgeries | 2.24 (0.90, 6.13) | 0.09 |
| CMV mismatch (D+/R-) | 1.20 (0.62, 2.25) | 0.57 |
| CAD |  |  |
| Age | 1.33 (0.91, 1.96) | 0.14 |
| BMI | 0.95 (0.63, 1.37) | 0.79 |
| Hypertension | 2.16 (1.36, 3.83) | 0.003 |
| Dyslipidemia | 2.39 (1.63, 3.58) | <0.001 |
| Diabetes mellitus | 1.45 (1.01, 2.09) | 0.043 |
| Current Smoking | 0.98 (0.66, 1.42) | 0.92 |
| LDL-C | 0.68 (0.44, 1.01) | 0.067 |
| eGFR | 0.98 (0.68, 1.43) | 0.91 |
| HbA1c | 1.55 (1.06, 2.35) | 0.028 |

Univariate logistic regression analysis of baseline characteristics including cardiovascular risk factors, transplant metrics and laboratory measurements with cardiac allograft vasculopathy and coronary artery disease. Abbreviations: BMI = Body Mass Index, CAD = Coronary Artery Disease, CAV = Cardiac Allograft Vasculopathy, CMV = Cytomegalovirus, eGFR = Glomerular Filtration Rate, HbA1c = HemoglobinA1c, LDL-C = Low-Density Lipoprotein Cholesterol, OR = Odds Ratio, SD = Standard Deviation.

**Figure S3: Prevalence of Coronary Artery Disease and Cardiac Allograft Vasculopathy Severity.**

The percentages of matched patients with CAD in the non-HTX cohort are on the left in yellow, while patients with CAV in the post-HTX cohort are on the right in red. Abbreviations: 1-VD = One Vessel Disease, 2-VD = Two Vessel Disease, 3-VD = Three Vessel Disease, CAD = Coronary Artery Disease, CAV = Cardiac Allograft Vasculopathy, CAV 0 = Not significant CAV 1 = Mild, CAV 2 = Moderate, CAV 3 = Severe, HTX = Heart Transplantation, CAD 0 = No Significant Coronary Artery Disease.
